# Assessing Knowledge, Preventive Practices, and Depression Among Chinese International Students and Local Korean Students in South Korea During the COVID-19 Pandemic: An Online Cross-Sectional Study

**DOI:** 10.3389/fpsyt.2022.920887

**Published:** 2022-06-21

**Authors:** Xiaoxu Jiang, Bo Zhao, Eun Woo Nam, Fanlei Kong

**Affiliations:** ^1^Centre for Health Management and Policy Research, School of Public Health, Cheeloo College of Medicine, Shandong University, Jinan, China; ^2^NHC Key Lab of Health Economics and Policy Research, Shandong University, Jinan, China; ^3^Department of Health Administration, Graduate School, Yonsei University, Wonju, South Korea; ^4^Yonsei Global Health Center, Yonsei University, Wonju, South Korea

**Keywords:** depression, preventive practices, PHQ-9, Chinese international students, South Korea, COVID-19

## Abstract

Depression among university students and international university students is an increasing problem globally. This study aimed to clarify the differences on the conditions and determinants of the knowledge, preventive practices and depression of the Chinese international students and local Korean students in South Korea during the COVID-19 pandemic. An online cross-sectional questionnaire including general demographic characteristics, COVID-19-related knowledge, preventive practice, and the Patient Health Questionnaire (PHQ-9) was applied from March 23 to April 22, 2020. A total of 533 university students (171 Chinese international students and 362 local South Korean students) were included in the study. The majority of both Chinese international students and local South Korean students had a good comprehension of COVID-19. Chinese international students in South Korea showed better preventive practice than local Korean students, while the proportion of moderate to severe depression of Chinese international students was relatively higher (28.07%) than that of local Korean students (22.38%). Determinants of depression of Chinese international students in South Korea were information satisfaction, likelihood of survival after infection, symptoms of a cough and feelings of discrimination, while for local Korean students were gender, educational level, family, suspected symptoms, self-assessed physical health status, COVID-19 detection, population contact history and online sources of information. These results could be used as a reference for decreasing the depressive symptoms among the university students.

## Introduction

The coronavirus disease (COVID-19) first appeared in Wuhan, China, spreading nationwide between December 2019 and early 2020, and then quickly to various countries (1). On January 30, 2020, the World Health Organization (WHO) declared the SARS-CoV-2 outbreak as a public health emergency of international concern, and on March 11, in Geneva, Switzerland, WHO Director-General Desmond Tan attended a press conference wherein he stated that the outbreak should be considered a pandemic (2). Starting in February 2020, South Korea experienced a sharp increase in confirmed cases (3). As of November 8, 2021, there were 126,713 and 5,696 cumulative confirmed cases and deaths, respectively, in China and 381,694 and 2,980 cumulative confirmed cases and deaths in South Korea, and the pandemic is reoccurring and circulating at multiple points. Knowledge and preventive practice are important to restrict the spread and avoid the related mental health problems during COVID-19 (4).

The main routes of transmission are air, droplets, aerosols, and, to a lesser extent, urine and feces (5). The study of knowledge, attitudes, and perceptions of COVID-19 in sub-Saharan Africa noted that, although most participants had adequate knowledge, attitudes were not always positive (6). The effect of control measures on COVID-19 transmission in South Korea confirms the importance of maintaining social distance and emphasizing personal hygiene, such as wearing masks, washing hands, and avoiding gatherings (7). Numerous studies showed that this pandemic leads to additional mental health problems, including anxiety, depression, insomnia, suicide, and self-injury (8–10).

Higher education levels were associated with possible depression and a higher frequency of mask wearing (11). Following preventive health behaviors may be safer for people and their health, It is also associated with lower depression, anxiety, and stress (12). Higher levels of psychological distress were associated with higher levels of fear. Moreover, if higher levels of psychological problems were demonstrated by those who identified as patients and individuals in isolation, then resilience played a key role in overcoming the psychological impact (13). A Malaysian study noted that introducing adequate, easy-to-follow precautions and standard operating procedures by university healthcare workers was associated with a reduction in their depression, anxiety, and stress (14). The use of online psychotherapy and the promotion of online social platforms to maintain social communication and relationships were significantly associated with increased physical, psychological, and social quality of life (QoL) and decreased depressive and anxiety symptoms as well as depression with anxiety symptoms during the COVID-19 (15).

During COVID-19 pandemic, a significant increase in psychological distress, fear, allostatic load, fatigue, loneliness, and worry was observed across different populations (16–18). During an outbreak, children are restricted to their homes without outdoor activities or interaction with friends. Hence, the negative impact on mental health can be more severe (19). COVID-19 led to significantly higher unemployment and suicide rates, and insecurity and uncertainty about the future deteriorated the psychological condition of migrant workers (20). There is a higher prevalence of anxiety, burnout, depression, and psychological distress among healthcare workers (21). COVID-19 infection negatively affects the psychological state of soccer players, with higher scores for depression, stress, and psychological distress among soccer players with higher income loss (22).

Although some studies explored the psychological adverse effects of COVID-19, the investigation of depression symptoms during the COVID-19 pandemic among the Chinese and Korean students had received little attention. According to the Korea Education Development Agency, among the total number of international students in South Korea, 67,030 were Chinese in 2020, accounting for 43.6% (23). The university students in South Korea, who are socially vulnerable, are more prone to depression during the COVID-19 pandemic (24). Although there is no nationwide lockdown in South Korea, strict control measures are implemented in public places, educational institutions, churches, and university campuses, where isolation and disconnection from society are a greater burden for young college students (25).

Concerning the international students in South Korea, a study of psychological problems in Korea showed that the prevalence of anxiety and depression among them was 39.6 and 49%, respectively (9). The dire situation during COVID-19 exacerbated the difficult situation for Chinese international students studying in South Korea. They faced isolation and discrimination because China was the first country to experience a pandemic. They were unable to return home and faced many problems such as online classes, difficulty in buying masks, and psychological issues on foreign campuses (26). Higher severity of depression may lower university students' QoL (27). This aggravates their depressive condition.

Although living in the same country, the international students and local students may face different situations and confront different mental health problems during the pandemic (28). However, to date, no study has examined the difference between the Chinese international students' and local Korean students' knowledge, preventive practice, and depression in South Korea during the pandemic. Thus, this study aimed to investigate the differences between knowledge, preventive practice, and depression among Chinese international students and local Korean students in South Korea, and identify the determinants of depression among them to provide policy references on preventive measures.

## Materials and Methods

### Data and Sample

#### Sampling Method

An online cross-sectional survey was used to collect data through an anonymous questionnaire in South Korea (during the COVID-19 pandemic). A total of 570 questionnaires were collected by snowball sampling, including many universities in South Korea. From March 23 to April 8, 2020, 180 responses from Chinese students and from March 23 to April 22, 2020, 390 responses from South Korean students were collected. Since the subjects were university students, 37 students who positively answered the employment question were excluded, leaving 533 students (171 Chinese international students and 362 South Korean students in South Korea). All respondents expressed their willingness to participate and claimed to understand the research background and purpose.

#### Data Collection Procedure

First, the English version of the COVID-19 questionnaire used by Wang et al. (29) and colleagues was adopted. Second, the required sample size was calculated using the G^*^Power 3.19 program. Based on the parameters of a two-sided test and χ^2^ test, a residual variance of 0.83, α probability = 0.05, and power = 0.95 for F tests and linear multiple regression analysis, the minimum total sample size was estimated to be 356.

With limited access to respondents due to social distancing policies in South Korea during the COVID-19 pandemic, an online cross-sectional survey was conducted. Before conducting the survey, the content of the questionnaire was verified through an online pre-survey of 15–30 students, to ensure that the questions were understandable, and the statements were appropriate. Researchers examined the questionnaires' reliability and the simplicity of the responses. Potential respondents were sent a specific link to participate. In total, with the help of collaborators and native Korean speakers, the questionnaire was placed on the Naver Form Tool survey platform in South Korea.

### Measurements

The questionnaire included two sections: (1) a questionnaire on COVID-19 and (2) the Patient Health Questionnaire-9 (PHQ-9). The first section comprised questions that covered (1) demographics and physical health data, (2) knowledge of COVID-19, and (3) preventive practices in the past 14 days.

### Description of the Variables

#### General Demographics

In this study, to reflect on the participants' demographic characteristics, the basic survey asked respondents about their gender, age, education level, marital status, family size, health insurance, chronic diseases, traveling status in the past 14 days, isolation, COVID-19 symptoms, and their self-assessed physical condition. The choices for education level were undergraduate and graduate and for marital status were single and married. Family size was divided into one person, two people, three to five people, and six or more people. Questions about medical insurance, chronic disease, traveling, self-quarantine, and symptoms were answered with yes or no. Regarding self-assessed physical condition, respondents were asked to choose from four levels: poor, fair, good, or very good.

#### Knowledge of COVID-19

Assessed knowledge of COVID-19 included transmission route, attention to updated information, information sources, concern for COVID-19, satisfaction with the information, confidence in diagnosis, infection probability, probability of survival after infection, concern for family members, feelings of being discriminated against, purchase face masks, and additional information about COVID-19.

#### Preventive Practices of COVID-19

The questionnaire included nine basic preventive practices. The responses to these questions corresponded to the degree to which a measure was practiced daily (1 = never do this and 5 = do this every day). The total score indicates the extent to which preventive practices were performed. The reliability (Cronbach's α) of the preventive practices of the COVID-19 scale in this study was 0.71 and the validity was 0.76.

#### Patient Health Questionnaire-9

Depressive symptoms were diagnosed according to the nine criteria for depression in the Diagnostic and Statistical Manual of Mental Disorders (DSM) published by the American Psychiatric Association (30). The options for each question in the PHQ-9 were (corresponding to the relative scores): not at all (0 points), occasionally (1 point), frequently (2 points), and almost every day (3 points). Respondents were divided into five groups according to their total score (31) (0–4, 5–9, 10–14, 15–19, and 20–27), corresponding to no depression, mild, moderate, moderately severe, and severe depression (32). The higher the score, the more severe the depression (33). The reliability of the PHQ-9 was 0.89, and its validity was 0.90 (34).

### Statistical Analysis

In this study, SPSS 24.0 (IBM Corp., Armonk, NY, US) was used to conduct the statistical analysis, and *p*-values < 0.05 were regarded as statistically significant. First, descriptive statistics, *t*-test, and chi-squared test were performed to compare each variable between the Chinese international and local students in South Korea. Second, stepwise linear regression was used to explore the determinants of depression among two student groups.

## Results

### General Characteristics

As shown in Table 1, there were significant differences between the two groups, regarding gender, age, educational level, marital status, family size, health insurance, self-quarantine, symptoms, and self-assessed physical condition. Among them, the female respondents were far higher than the male respondents, specifically, among the Chinese international students. The average age of Chinese international students was 24.02 ± 4.14 years, while that of local South Korean students was 22.13 ± 3.11 years. Moreover, the proportion of graduate students, married respondents, families with two people, and medical insurance of Chinese international students was higher. In contrast, most Chinese international students in South Korea (83.04%) have experienced self-isolation, while more than 90% of local respondents stated that they have not. Chinese international students showed better physical condition and fewer suspected symptoms (10.53%) than local students (32.32%).

**Table 1:** Demographics characteristics of the respondents.

	**Chinese international students**	**Local South Korean**			
**Variables**	**in South Korea (*n =* 171)**	**students (*n =* 362)**	**Total (*n =* 533)**	**t/χ^2^**	***P***
	***N*** **(%)**	***N*** **(%)**	***N*** **(%)**		
**Age**					
Mean ± S.D	24.02 ± 4.14	22.13 ± 3.11	22.74 ± 3.58	2.075^a^	0.040
**Gender**					
Male	57 (33.33)	154 (42.54)	211 (39.59)	4.118^b^	0.042
Female	114 (66.67)	208 (57.46)	322 (60.41)		
**Educational level**					
Undergraduate	98 (57.31)	334 (92.27)	432 (81.05)	92.396^b^	<0.001
Graduate	73 (42.69)	28 (7.73)	101 (18.95)		
**Marital status**					
Single	159 (92.98)	358 (98.90)	517 (97.00)	13.944^b^	<0.001
Married	12 (7.02)	4 (1.10)	16 (3.00)		
**Family size**					
1 member	9 (5.26)	38 (10.50)	47 (8.82)	17.286^b^	0.001
2 members	24 (14.04)	17 (4.70)	41 (7.69)		
3–5 members	134 (78.36)	295 (81.49)	429 (80.49)		
6 members or more	4 (2.34)	12 (3.31)	16 (3.00)		
**Medical insurance**					
No	28 (16.37)	128 (35.36)	156 (29.27)	20.220^b^	<0.001
Yes	143 (83.63)	234 (64.64)	377 (70.73)		
**Chronic diseases**					
No	159 (92.98)	327 (90.33)	486 (91.18)	1.015^b^	0.314
Yes	12 (7.02)	35 (9.67)	47 (8.82)		
**Traveled abroad**					
No	166 (97.08)	359 (99.17)	525 (98.50)	2.177^b^	0.140
Yes	5 (2.92)	3 (0.83)	8 (1.50)		
**Self-quarantine**					
No	29 (16.96)	328 (90.61)	357 (66.98)	284.827^b^	<0.001
Yes	142 (83.04)	34 (9.39)	176 (33.02)		
**Several symptoms**					
No	153 (89.47)	245 (67.68)	398 (74.67)	29.167^b^	<0.001
Yes	18 (10.53)	117 (32.32)	135 (25.33)		
**Specific symptoms**					
Fever (yes)	3	5	8	1.425^b^	0.233
Chills (yes)	4	9	13	4.324^b^	0.038
Headache (yes)	5	58	63	19.117^b^	<0.001
Muscle pain (yes)	5	29	34	12.058^b^	0.001
Cough (yes)	4	35	39	9.200^b^	0.002
Dyspnea (yes)	8	20	28	3.948^b^	0.045
Dizziness (yes)	2	22	24	10.840^b^	0.001
Nasal cold (yes)	7	38	45	6.161^b^	0.013
Laryngitis (yes)	9	22	31	0.141^b^	0.708
Nausea (yes)	3	19	24	9.307^b^	0.002
**Self-assessed physical condition**					
Poor	1 (0.58)	7 (1.93)	8 (1.50)	20.712^b^	<0.001
Fair	17 (9.94)	92 (25.41)	109 (20.45)		
Good	85 (49.71)	154 (42.54)	239 (44.84)		
Very good	68 (39.77)	109 (30.11)	177 (33.21)		
**COVID−19 detection**					
Yes	11 (6.4)	7 (1.9)	18 (3.4)	7.204^b^	0.007
No	160 (93.6)	355 (98.1)	515 (96.6)		
**Patient contact history**					
Yes	2 (6.4)	8 (6.4)	10 (6.4)		
No	12 (6.4)	52 (6.4)	64 (6.4)	6.840^b^	0.030
Not clear	157 (6.4)	302 (6.4)	459 (6.4)		

a *t-test,*

b *χ^2^*.

### Knowledge of COVID-19

Table 2 shows the participants' knowledge of COVID-19, with about 98.12% knowing that the virus can be transmitted by droplets, 79.17% agreeing that transmission is possible through objects touched by the infected person, and only half (50.84%) agreeing that it can be transmitted through the air. Most students in both groups (more than 90%) were interested in the latest information on infections, cures, and deaths. Their main sources of information are the Internet, TV, and family, and they rarely get information from other channels.

**Table 2:** Differences in knowledge of COVID-19.

	**Chinese international students**	**Local South Korean**			
**Variables**	**in South Korea (*n =* 171)**	**students (*n =* 362)**	**Total (*n =* 533)**	**χ^2^**	***P***
	***N*** **(%)**	***N*** **(%)**	***N*** **(%)**		
**Route of transmission**					
Droplets (agree)	171 (100)	352 (97.24)	523 (98.12)	4.814	0.090
Objects (agree)	145 (84.80)	277 (76.52)	422 (79.17)	5.144	0.076
Air (agree)	116 (67.84)	155 (42.82)	271 (50.84)	29.455	<0.001
**Updated information**					
Infections (yes)	168 (98.25)	352 (97.24)	520 (97.56)	0.163	0.687
Deaths (yes)	169 (98.83)	345 (95.30)	514 (96.44)	4.202	0.040
Recoveries (yes)	163 (95.32)	323 (89.23)	486 (91.18)	5.366	0.021
**Information sources**					
Internet (yes)	169 (98.83)	338 (93.37)	507 (95.12)	7.462	0.006
TV (yes)	72 (42.11)	205 (56.63)	277 (51.97)	9.816	0.002
Radio (yes)	11 (6.43)	7 (1.93)	18 (3.38)	7.204	0.007
Newspaper (yes)	14 (8.19)	14 (3.87)	28 (5.25)	4.354	0.037
Family (yes)	54 (31.58)	86 (23.76)	140 (26.27)	3.669	0.055
Other (yes)	7 (4.09)	13 (3.59)	20 (3.75)	0.081	0.776
**Concern about the disease**					
Never	3 (1.75)	14 (3.87)	17 (3.19)	43.576	<0.001
Rarely	8 (4.68)	40 (11.05)	48 (9.01)		
Sometimes	35 (20.47)	110 (30.39)	145 (27.20)		
Often	48 (28.07)	131 (36.19)	179 (33.58)		
Everyday	77 (45.03)	67 (18.51)	144 (27.02)		
**Satisfaction with the information**					
Very dissatisfied	2 (1.17)	3 (0.83)	5 (0.94)	65.337	<0.001
Dissatisfied	12 (7.02)	110 (30.39)	121 (22.70)		
Satisfied	115 (67.25)	228 (62.98)	343 (64.35)		
Very satisfied	42 (24.56)	21 (5.80)	63 (11.82)		
**Confidence about being diagnosed**					
Do not believe	7 (4.09)	4 (1.10)	11 (2.06)	63.354	<0.001
Hard to believe	42 (24.56)	15 (4.14)	57 (10.69)		
To some extent	71 (41.52)	252 (69.61)	323 (60.60)		
Very believe	30 (17.54)	58 (16.02)	88 (16.51)		
Not clear	21 (12.28)	33 (9.12)	54 (10.13)		
**Infection probability**					
Very low	17 (9.94)	108 (29.83)	125 (23.45)	36.683	<0.001
Low	79 (46.20)	95 (26.24)	174 (32.65)		
High	58 (33.92)	114 (31.49)	172 (32.27)		
Very high	1 (0.58)	5 (1.38)	6 (1.13)		
Not clear	16 (9.36)	40 (11.05)	56 (10.51)		
**The likelihood of survival after infection**					
Very low	2 (1.17)	12 (3.31)	14 (2.63)	9.132	0.054
Low	2 (1.17)	9 (2.49)	11 (2.06)		
High	72 (42.11)	146 (40.33)	218 (40.90)		
Very high	85 (49.71)	151 (41.71)	236 (44.28)		
Not clear	10 (5.85)	44 (12.15)	54 (10.13)		
**Concern about family members**					
Not at all	27 (15.79)	16 (4.42)	43 (8.07)	28.536	<0.001
Little	71 (41.52)	191 (52.76)	262 (49.16)		
Very worried	68 (39.77)	123 (33.98)	191 (35.83)		
Not clear	5 (2.92)	32 (8.84)	37 (6.94)		
**Whether they feel their country is discriminated against**					
Yes	65 (38.01)	156 (43.09)	221 (41.46)	25.932	<0.001
No	87 (50.88)	111 (30.66)	198 (37.15)		
Not clear	19 (11.11)	95 (26.24)	114 (21.39)		
**Whether purchased face masks**					
Yes	26 (15.20)	253 (69.89)	279 (52.35)	139.228	<0.001
No	145 (84.80)	109 (30.11)	254 (47.65)		
**For additional information on COVID-19**					
Yes	160 (93.57)	206 (56.91)	366 (68.67)	72.552	<0.001
No	11 (6.43)	156 (43.09)	167 (31.33)		

Chinese international students showed high levels of concern about COVID-19, with 45% worrying about it daily. Among the local South Korean students, 30% were dissatisfied with the information currently provided, compared to <10% of Chinese international students. Some showed a degree of confidence about getting infected (41.52% for Chinese international and 69.61% for local students) and 30% of respondents in each group said they were very likely to be infected, but most also thought they had a good chance of survival. Some from the two groups were significantly worried about their families (39.77% for Chinese international and 33.98% for local students). Nearly half of the Chinese international students surveyed felt there was no discrimination regarding their country, compared to 30% of local South Korean students. As many as 85% of Chinese international students stated that they could not buy masks for reasons such as the lack of South Korea's national health insurance and the shortage. A small number of local students (30.11%) were unable to buy masks due to long queues and lack of availability.

### Differences in the Type of Information They Want to Obtain

**Figure 1:**
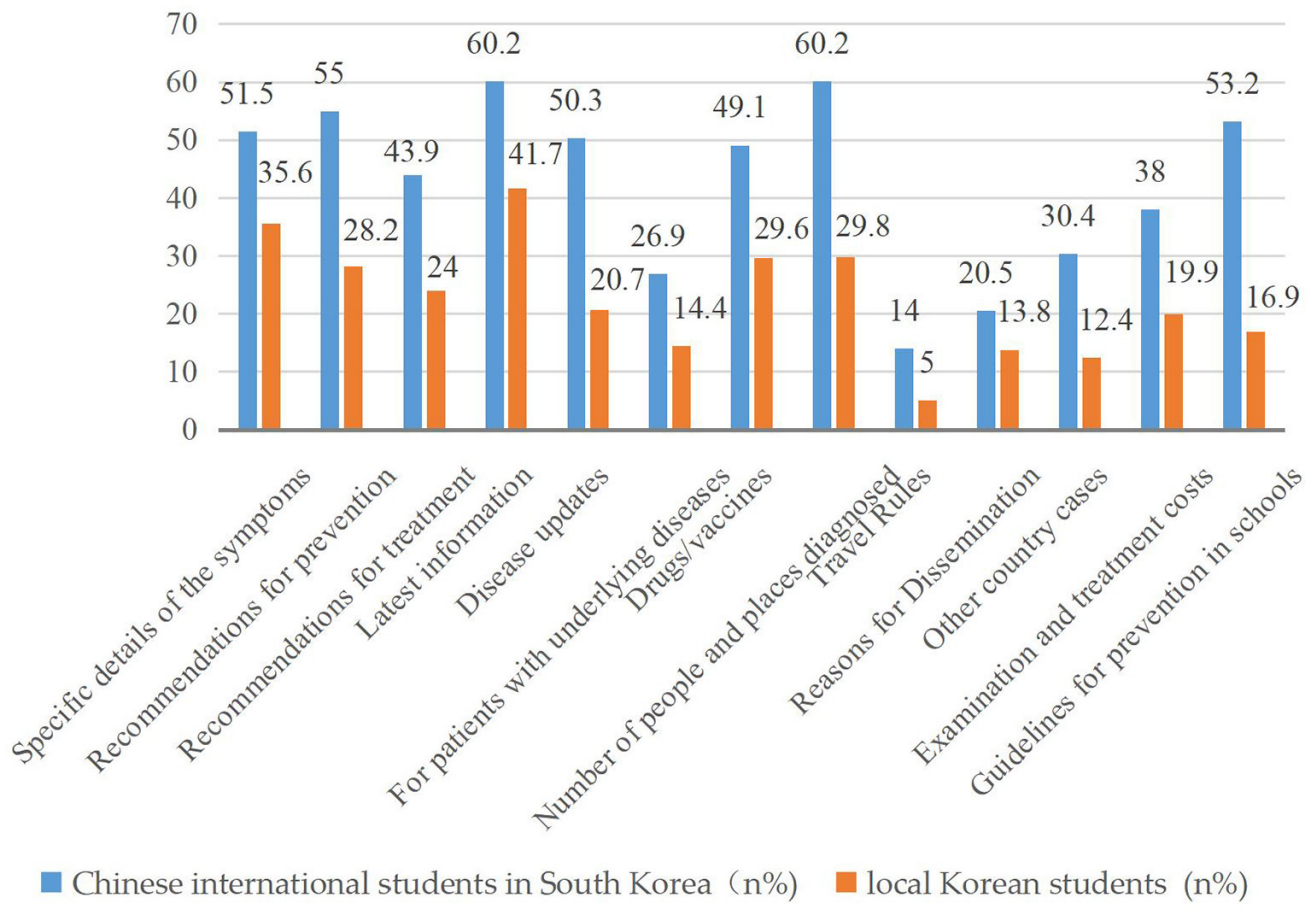
Type of information expected between Chinese international students and local Korean students in South Korea.

Figure 1 shows the types of information that Chinese international students and local students wanted to obtain. The former were eager to access more information. More than half of the Chinese international students wanted to know specific details of symptoms, advice on prevention, up-to-date information, the number of people and places diagnosed, and the school's prevention code. The top five types of information in demand among local Korean students were up-to-date information, specific details of symptoms, availability, and effectiveness of vaccines/drugs, number of people and places infected, and prevention advice.

### Differences in Preventive Practices Between the Two Groups

Table 3 shows the preventive practices of Chinese international students and local Korean students in South Korea. The average total score of the Chinese international students (38.67%) was slightly higher than that of local Korean students (33.80%). There were statistically significant differences regarding the eight preventive behaviors between the two groups. This included when coughing and sneezing, wearing masks regardless of the presence or absence of symptoms, washing hands immediately after coughing, rubbing the nose, sneezing, washing hands after touching contaminated objects, avoiding public transportation, avoiding elevators, sitting in one row while having a meal, and avoiding meeting more than 10 people.

**Table 3:** Preventive practices taken against COVID-19 (Mean ± S.D).

**Variables**	**Chinese international students**		**Local South Korean**				**t**
	**in South Korea (*****n =*** **171)**		**students (*****n =*** **362)**		**Total (*****n =*** **533)**		
	**Mean ±S.D**.	**95% Conf. interval**	**Mean ±S.D**.	**95% Conf. interval**	**Mean ±S.D**.	**95% Conf. interval**	
1.Covering mouth when coughing and sneezing	4.67 ± 0.80	4.55–4.79	4.46 ± 0.93	4.36–4.55	4.53 ± 0.89	4.45–4.60	2.63^**^
2. Wearing mask regardless of the presence or absence of symptoms	4.32 ± 0.80	4.20–4.44	3.16 ± 1.46	3.01–3.31	3.53 ± 1.39	3.42–3.65	9.73^***^
3. Washing hands with soap and water	4.84 ± 0.44	4.78–4.91	4.75 ± 0.60	4.69–4.82	4.78 ± 0.55	4.74–4.83	1.72
4. Washing hands immediately after coughing, rubbing nose, or sneezing	4.18 ± 1.05	4.02–4.33	3.62 ± 1.20	3.50–3.75	3.80 ± 1.18	3.70–3.90	5.14^***^
5. Washing hands after touching contaminated objects	4.93 ± 0.39	4.87–4.99	4.72 ± 0.67	4.65–4.78	4.78 ± 0.60	4.73–4.84	3.89^***^
6. Avoiding public transportation	4.74 ± 0.67	4.64–4.84	4.40 ± 0.96	4.30–4.50	4.51 ± 0.89	4.43–4.59	4.11^***^
7. Avoiding elevators	3.44 ± 1.40	3.23–3.66	2.33 ± 1.49	2.17–2.48	2.69 ± 1.55	2.55–2.82	8.41^***^
8. Sitting in one row while having a meal	2.80 ± 1.70	2.55–3.06	2.11 ± 1.41	1.97–2.26	2.33 ± 1.54	2.20–2.46	4.95^***^
9. Avoiding meeting more than 10 people	4.74 ± 0.89	4.61–4.88	4.25 ± 1.20	4.12–4.37	4.41 ± 1.13	4.31–4.50	4.83^***^
Total score	38.67 ± 4.62	37.9–39.3	33.80 ± 5.32	33.2–34.3	35.36 ± 5.59	34.8–35.8	10.258^*^

* *p < 0.05*.

** *p < 0.01*.

*** *p < 0.001*.

### Results and Analysis of Depressive Conditions

A chi-square test was used to determine the difference in the level of depressive symptoms between Chinese international students and local Korean students (Table 4). In this study, depressive symptoms were classified as 0–4 (no depression), 5–9 (mild depression), 10–14 (moderate depression), 15–19 (moderate to severe depression), and 20–27 (severe depression). As shown in Table 4, the differences between the two groups were statistically significant (**χ**^2^= 11.224, p < 0.05), and Chinese international students were slightly more depressed.

**Table 4:** Difference in depressive symptoms between the Chinese international students and local Korean students.

	**Chinese international students**	**Local South Korean**		
**PHQ-9**	**in South Korea (*n =* 171)**	**students (*n =* 362)**	**Total (*n =* 533)**	**t/χ^2^**
	***N*** **(%)**	***N*** **(%)**	***N*** **(%)**	
Total score^a^	7.19 ± 5.34	5.99 ± 5.46	6.38 ± 5.45	*t* = 2.38^*^
0–4 (non-depressed)	60 (35.09)	182 (50.28)	242 (45.40)	χ^2^ = 11.224^*^
5–9 (Mild)	63 (36.84)	99 (27.35)	162 (30.39)	
10–14 (Moderate)	31 (18.13)	50 (13.81)	81 (15.20)	
15–19 (Moderately severe)	11 (6.43)	22 (6.08)	33 (6.19)	
20–27 (Severe)	6 (3.51)	9 (2.49)	15 (2.81)	

a *The total score of the PHQ-9 is mean ± SD*.

* *p < 0.05*.

Linear stepwise regression was used to explore the factors affecting the depressive status of the Chinese international students (Table 5) and the local South Korean students (Table 6), separately. *P*-values < 0.05 were regarded as statistically significant. All variables that were statistically significant in Tables 1–3 were all included in Tables 5, 6. These included age, gender, education, marital status, family size, health insurance, self-isolation, general symptoms and specific symptoms, self-assessed physical health status, means of transmission, updated information, information sources (Internet, TV, radio, newspapers, family), level of satisfaction with information, COVID-19 testing, patient contact history, confidence in confirmed diagnosis, probability of infection and surviving infection, level of concern about family members, discrimination, mask purchase, additional information, and eight preventive measures. The results in Table 5 showed that among Chinese international students, perceived discrimination in their country, probability of survival after infection, cough symptoms, and satisfaction with information were statistically significantly related to respondents' depression. Regarding the local South Korean students, the results in Table 6 illustrated that gender, education level, family size, self-assessed physical health status, symptoms of nausea and dyspnea, nucleic acid testing, history of patient exposure, and receipt of outbreak-related information from family members and the Internet were statistically significantly related to respondents' depression.

**Table 5:** Stepwise regression analysis of the factors of depression due to COVID-19 among Chinese international students in South Korea.

**Dependent variable**	**Independent variables**	**β**	**S.E.**	**β'**	***t***	***P***
PHQ-9 scores	Constant	20.231	2.903		6.970	0.000
	Discrimination psychological	−1.234	0.406	−0.217	−3.037	0.003
	The likelihood of survival after infection	−1.377	0.506	−0.194	−2.721	0.007
	Symptoms of a cough	7.468	2.504	0.212	2.983	0.003
	Satisfaction With the information	−1.121	0.481	−0.167	−2.328	0.021

*S.E., Standardized Error*.

**Table 6:** Stepwise regression analysis of the factors of depression due to COVID-19 among local South Korean students.

**Dependent variable**	**Independent variables**	**β**	**S.E.**	**β'**	***t***	***P***
PHQ-9 scores	Constant	19.749	3.007		6.568	0.000
	Gender	1.168	0.533	0.106	2.189	0.029
	Educational Level	−3.264	1.052	−0.160	−3.101	0.002
	Family size	−0.906	0.418	−0.111	−2.165	0.031
	Self-assessed physical condition	−1.649	0.339	−0.240	−4.866	0.000
	Symptoms of nausea	2.454	1.231	0.100	1.994	0.047
	Symptoms of dyspnea	8.454	3.575	0.115	2.365	0.019
	COVID-−19 detection	5.556	1.911	0.140	2.908	0.004
	Patient contact history	−1.227	0.586	−0.100	−2.095	0.037
	Get information from family	1.239	0.621	0.097	1.994	0.047
	Get information on the internet	−4.197	1.875	−0.106	−2.238	0.026

*S.E., Standardized Error*.

## Discussion

### Principal Findings and Comparison With Other Studies

This study used univariate and multiple linear regression analyses to clarify the relationship between knowledge, preventive practices, and depression among Chinese international students and local Korean students in South Korea. The results revealed that the determinants of depression affecting the two groups were different. Targeted measures should be taken to reduce depression.

### Demographic Characteristics of Chinese International Students and Local South Korean Students

Most international students in the sample reported having insurance, However, it was international student insurance rather than South Korean national health insurance. This resulted in Chinese international students in South Korea being unable to buy masks at the beginning of the outbreak due to the restrictions of the national health insurance (35). Most of them in the sample were self-isolated, while the vast majority of local South Korean students did not experience self-isolation. This is because South Korea is a narrow and densely populated country, and the government did not take measures (36) for widespread self-isolation of the population in the early stages of the COVID-19 pandemic but only isolated and treated confirmed patients. The self-assessed physical health of the local South Korean students in the sample was less favorable overall, consistent with the findings of Sun Jung Kim's study (37), which found that Chinese students in South Korea had a high level of self-assessed health and cultural adaptation.

### Knowledge and Preventive Practice of Chinese International Students and Local South Korean Students

In this study, both student groups were concerned about COVID-19 and showed a good level of knowledge, which is consistent with the results of other studies (38, 39). The Internet is the main information source, and they are satisfied with the information currently provided. This is because the central and local public health authorities in South Korea provide daily updates of all laboratory-confirmed COVID-19 cases on their web pages including information on the number of national and local cases, age, sex, symptoms, date of onset of symptoms, source, date of exposure, and location of the infection (within or outside Korea) (40). Chinese international students generally perceive themselves as having a low probability of being infected, which is also linked to the widespread self-isolation of international students (4). The local students believe that the higher probability of infection is related to the surge in the number of infections in Korea at the beginning of the pandemic (41). Chinese students have significantly better preventive practice behaviors, specifically, Chinese international students are particularly better at wearing masks and washing their hands, which also suggests that they were more afraid of being infected and more vulnerable to the effects of the pandemic (42).

### Differences in Depression Between Chinese International Students and Local South Korean Students

Depression rates were fairly high for both student groups, since the Chinese international students in South Korea have a higher rate (18.13%) of moderate depression than local students (13.81%), and a slightly higher rate (3.51%) of severe depression than local students (2.49%). This result is consistent with many local South Korean studies. Jimhee's survey showed that the prevalence of depression among the general population of Korea at the beginning of the pandemic was 19%(43), Lee's study on adolescents in Daegu, South Korea, during the COVID-19 pandemic showed that 19.8% and 12.3% of students experienced depression and anxiety, respectively (44), Hoo's study of adults aged 20 to 49 in Chungnam Province, South Korea, during the COVID-19 pandemic found that 18.8% of participants had symptoms of depression (45). In Kim's study of 180 nurses during the pandemic, 30.6% had moderate or higher levels of depression (46). Kim and Lee's study found that depression's prevalence diagnosed in South Korea in 2016 was 3%, with a slightly higher prevalence among males (3.1%) than females (2.9%) among those aged 18–29 years (47), indicating the pandemic may increase the prevalence of the depression.

Satisfaction with information was associated with depression among Chinese international students in South Korea in this sample. Specifically, students who showed higher satisfaction were less likely to be depressed. This also confirmed the importance of the provision of accurate and truthful information by the government during a pandemic. Kim's research during the Korean pandemic indicated that useful information related to COVID-19 could help prevent infection as well as promote anxiety and fear, leading to negative behavior (47). Another factor that influenced depression among the Chinese international students in the sample was the likelihood of survival after infection. This is clear proof that international students are significantly worried about the pandemic (38). The feeling of being discriminated against was a predictor of depression among Chinese international students. This was consistent with the findings of a study by Li on Chinese and American students, who noted that the former live in fear of discrimination in the US, which significantly affected their emotions and self-esteem (48). Therefore, greater attention should be paid to international students' mental health to help them build self-esteem and self-confidence.

For the local South Korean students in the sample, gender and education level were predictors of depression. This was consistent with the findings of Won, who found higher rates of depression among women in a South Korean community survey (49). Self-assessed physical health status was associated with depression among local South Korean students. The result was similar to the findings of an online survey on 400 South Korean people conducted by Hye during the pandemic from March to June 2020, which found a statistically significant association between mental and physical health (25). Another noteworthy factor affecting depression among local South Korean students was the information source, mainly the Internet. South Korea had done a good job of disclosing all information about COVID-19 to the public openly and transparently, holding detailed press conferences twice a day since the first COVID-19 case was diagnosed, and full disclosure promotes public trust and support for the government (50). Alwin noted that the South Korean government's transparency in sharing information about the main features of the outbreak and the standard procedures to be followed helped to combat the spread of rumors related to the outbreak (51). Information from families and family size could influence depression among local South Korean students, indicating the importance in their minds (52). Finally, nucleic acid testing and patient exposure history were correlated with depression among local South Korean students, which showed the importance of correct disclosure of information about COVID-19 (53).

Finally, there are two factors to be concerned about. First, the result showed that symptoms affected the depression among local South Korean and Chinese international students in South Korea. Symptoms of COVID-19 vary across patients, but the most common clinical symptoms include fever, malaise, cough, sputum, and shortness of breath at all stages of the disease, along with less common symptoms such as sore throat, headache, confusion, hemoptysis, shortness of breath and chest tightness. Moreover, minor symptoms include nausea, vomiting, diarrhea, and gastrointestinal complications. Therefore, it is difficult to identify the suspected COVID-19 symptoms because they are diverse, and for university students, the presence of a symptom adds to their psychological burden (54). Second, this study could not directly prove that personal precautions (wearing masks, washing hands regularly, and so on) would affect depression in both student groups. Christoph's findings also indicated that there is little evidence that public health measures were associated with direct mental health impairment (55). Wendy's research at American University also found that students' perceptions of wearing masks varied (56). While one study in Hong Kong concluded that the insecurity associated with wearing and reusing masks could increase the participants' psychological burden (57). A study conducted in mainland China among children and adolescents revealed that the frequency of wearing a mask and the duration of exercise were associated with mental health (58).

### Implications

The South Korean government is supposed to continue its efforts and support student development during the outbreak. For the international students in South Korea, first, as a large group, the government should remove the restriction on buying masks from national insurance. Second, transparent, open, and truthful information should be provided to international students. Third, attention should be paid to international students' mental health, providing them warmth and care in a timely manner so that they can build their self-esteem and self-confidence. Fourth, psychiatrists could use telemedicine for psychotherapy and the continuation of interpersonal therapy (59). Regarding local South Korean students, first, the government should pay attention to the mental health status of university students of different genders and educational levels. Second, measures to lower the history of outside contact should be undertaken. Third, attention should be paid to physical health and improving university students' physical fitness in South Korea. Fourth, the family's role in preventing infection and caring for patients should not be overlooked. Fifth, the Internet should be used as a positive guide to deliver accurate information. Finally, the testing speed of COVID-19 should be accelerated. Clinicians should use the sequential model which integrates psychotherapy and pharmacotherapy to prevent the recurrence of depression (60).

### Limitations and Future Research

This study had several limitations. First, it was conducted online, which may cause issues such as the uncertainty of the respondents' attitude and seriousness when completing it. Moreover, the self-assessed physical health status may not be the same as the one judged by professionals. Second, this study used snowball sampling and the sample size is not huge. Hence, the sample may not be representative of the general population. Third, the study would be more accurate if the comparison was between similar populations, with the only difference being nationality. It would be more informative to include the questionnaire used to assess the general demographics, knowledge of COVID-19, and preventive practices. Fourth, anxiety was not measured and anxiety level should be considered as a potential confounding variable in future studies. Besides, this study did not assess the factors that are associated with depression among university students such as the frustration due to loss of daily life and study interruptions, pre-existing depression, anxiety, and social support (61). Despite these limitations, this study provides useful information on the knowledge, prevention practices, and depression status of Chinese international and local Korean students in South Korea who experienced the most severe moments of the COVID-19 outbreak. It could serve as an evidence-based reference for providing psychological help to local and international students during the pandemic of infectious diseases.

## Conclusions

This study investigated the conditions and determinants of knowledge, preventive behaviors, and depression among Chinese international and local students living in South Korea during the early stage of COVID-19 pandemic. The results revealed that both student groups had good knowledge of COVID-19. Chinese international students had better preventive practice and showed higher levels of depression. Information satisfaction, the likelihood of survival after infection, symptoms of a cough, and feelings of discrimination were the determinants of depression among Chinese international students. Gender, educational level, family, suspected symptoms, self-assessed physical health status, COVID-19 detection, population contact history, and online sources of information were the determinants of the local students. The data may serve as a preliminary study on lowering the negative impact of COVID-19 on the mental health of the international and native students in a country and can be used as a guide for further studies in different regions.

## Data Availability Statement

The raw data supporting the conclusions of this article will be made available by the authors, without undue reservation.

## Ethics Statement

The studies involving human participants were reviewed and approved by Chair, Yonsei University Mirae Institutional Review Board. Affiliation: College of Health Sciences/Health Administration. The patients/participants provided their written informed consent to participate in this study.

## Author Contributions

Conceptualization: EN and BZ. Methodology, software, and data curation: BZ. Validation: FK, EN, and BZ. Formal analysis and writing—original draft preparation: XJ. Investigation and resources: FK and BZ. Writing—review and editing and supervision: FK and EN. All authors have read and agreed to the published version of the manuscript.

## Funding

This study was supported and funded by the National Natural Science Foundation of China (No. 71804094), China Postdoctoral Science Foundation (No. 2016M592161).

## Conflict of Interest

The authors declare that the research was conducted in the absence of any commercial or financial relationships that could be construed as a potential conflict of interest.

## Publisher's Note

All claims expressed in this article are solely those of the authors and do not necessarily represent those of their affiliated organizations, or those of the publisher, the editors and the reviewers. Any product that may be evaluated in this article, or claim that may be made by its manufacturer, is not guaranteed or endorsed by the publisher.
