# Supplementary material for: Assessing Knowledge, Preventive Practices, and Depression Among Chinese International Students and Local Korean Students in South Korea During the COVID-19 Pandemic: An Online Cross-Sectional Study
Source: Front Psychiatry. 2022 Jun 21;13:920887. doi: 10.3389/fpsyt.2022.920887 (PMC9258509; doi:10.3389/fpsyt.2022.920887)
Supplement: Supplementary file 1 [file Table_1.DOC]

**A study on the impact of the COVID-19 epidemic on the living condition of university students**

Hello, thank you for participating in our research during your busy schedule.

This survey was conducted to understand students' knowledge, preventive practices and depression related to COVID-19.

This survey is filled in anonymously. The information will be kept strictly confidential and used for scientific research only!

Your opinion is quite valuable to us, so please fill out the answer carefully.

Thank you for your assistance and good health!

Part A

1. Gender

Male

Female

2. Year of Birth

3. Education

University: Bachelor

University: Master or PhD

4. Residential area during the COVID-19 outbreak Please specify City (County)

5. Marital Status

Single

Married"

6. Employment Status

Student

Employed

Unemployed (No Work)

Other

7.Number of household

1 person

2 persons

3-5persons

6 persons or more

8. Have you traveled outside of your residential country in the past 14 days?

No

Yes

9.Please specify the countries you’ve visited past 14 days

Part B

1. Symptoms of body discomfort in the past 14 days (Multiple answer)？

No

Yes

2.Specific symptoms

Fever (yes or no)

Chills (yes or no)

Headaches (yes or no)

Myalgia (yes or no)

Cough (yes or no)

Difficulty Breathing (yes or no)

Dizziness (yes or no)

Nasal cold (yes or no)

Laryngitis (yes or no)

Nausea (yes or no)

3. Were you tested for COVID-19 in the past 14 days?

No

Yes

4. Were you under quarantine by health authority in the past 14 days?

No

Yes

5. Please self-rate your current health status

Very poor

Poor

Fair

Good

Very good

6. Do you have medical insurance from private sector?

No

Yes

7. Do you suffer from a chronic illness diagnosed by physician?

No

Yes

8. Have you ever experienced self-quarantine?

No

Yes

9. Have you directly or indirectly contacted patients suffering from COVID-19?

No

Yes

Don't know

Part C

1. Does the COVID-19 transmit through Droplets?

Agree

Disagree

Not clear

2. Does the COVID-19 transmit through Contact via contaminated objects？

Agree

Disagree

Not clear

3. Does the COVID-19 transmit through Air?

Agree

Disagree

Not clear

4. How satisfy you are with the amount of health information available about COVID-19？

Very Dissatisfied

Dissatisfied

Satisfied

Very satisfied

Not clear

5. Have you heard of the following… ？

[a. Number of confirmed cases infected by COVID-19]

Heard

Not heard/Don't know

[b. Number of deaths infected by COVID-19]

Heard

Not heard/Don't know

[c. Number of recovered cases infected by COVID-19]

Heard

Not heard/Don't know

6. How do you mainly get health information?

Internet

TV

Radio

Newspaper

Family member

Other

7. How do you trust in your own hospital to diagnose or recognize COVID-19？

Not at all confident

Not very confident

Somewhat confident

Very confident

Not clear

8. Please rate your likelihood of Contracting COVID-19 during the current outbreak.

Not likely at all

Not very likely

Somewhat likely

Very likely

Not clear

9. Please rate your likelihood of Surviving COVID-19 if infected.

Not likely at all

Not very likely

Somewhat likely

Very likely

Not clear

10. Please rate your concerns about other family members getting COVID-19.

Not worried at all

Not very worried

Very worried

Not applicable

Not clear

11. Do you feel that you are being discriminated by other countries due to the outbreak of COVID-19？

Yes

No

Not clear

12. Have you ever bought masks due to the outbreak of COVID-19?

Yes

No

13. Do you have any opinion (or complain) for buying masks?

14.Do you feel that too worry or anxiety has been made about COVID-19

Never

Rarely

Sometimes

Often

Everyday

Part D

1. Do you Covering mouth when coughing and sneezing in the past 14 days

Never

Sometime

Most of time

Occasional

Always

2. Do you Avoid public transportation in the past 14 days

Never

Sometime

Most of time

Occasional

Always

3. Do you Washing hands with soap and water in the past 14 days

Never

Sometime

Most of time

Occasional

Always

4. Do you Washing hands immediately after coughing, rubbing nose or sneezing in the past 14 days

Never

Sometime

Most of time

Occasional

Always

5. Do you Wearing mask regardless the presence or absence of symptoms in the past 14 days

Never

Sometime

Most of time

Occasional

Always

6. Do you Washing hands after touching contaminated objects in the past 14 days

Never

Sometime

Most of time

Occasional

Always

7. Do you Avoid using elevator in the past 14 days

Never

Sometime

Most of time

Occasional

Always

8. Do you Sit in one row while having meal in the past 14 day

Never

Sometime

Most of time

Occasional

Always

9. Do you Avoid meeting with more than 10 people in the past 14 days

Never

Sometime

Most of time

Occasional

Always

Part E

1. Would you like to receive additional information about COVID-19?

Yes

No

2. I would like to receive additional information about COVID-19 on...

Specific details of the symptoms (yes or no)

Recommendations for prevention (yes or no)

Recommendations for treatment (yes or no)

Latest information (yes or no)

Disease updates (yes or no)

For patients with underlying diseases (yes or no)

Drugs/vaccines (yes or no)

Number of people and places diagnosed (yes or no)

Travel Rules (yes or no)

Reasons for Dissemination (yes or no)

Other country cases (yes or no)

Examination and treatment costs (yes or no)

Guidelines for prevention in schools (yes or no)

PHQ-9

1. I seemed have no interest or pleasure in doing work or leisure activities

not at all

occasionally

frequently

almost every day

2. I felt depressed and hopeless

not at all

occasionally

frequently

almost every day

3. I found it difficult to fall asleep or keep sleeping, or slept too much suddenly

not at all

occasionally

frequently

almost every day

4. I felt tired or had little energy.

not at all

occasionally

frequently

almost every day

5. I tended to have no appetite or overeat

not at all

occasionally

frequently

almost every day

6. I felt that I have no confidence. / I felt like I am a failure and let myself or my family down

not at all

occasionally

frequently

almost every day

7. It was difficult to concentrate on such things as reading newspapers or watching television

not at all

occasionally

frequently

almost every day

8. When someone was watching me, I felt like moving or talking slowly. On the contrary, It was more active than usual, too restless or impetuous

not at all

occasionally

frequently

almost every day

9. I thought that it is better to die or that I would harm myself anyway

not at all

occasionally

frequently

almost every day
